# Relationship between seawater temperature, brain GnRH-like peptide expression, and gonadal development in wild bigfin reef squid (*Sepioteuthis lessoniana*)

**DOI:** 10.1186/s40659-025-00626-1

**Published:** 2025-07-02

**Authors:** Umina Kubo, Lee Jaewoo, Ryosuke Murata, Takashi Aoshima, Yuji Mushirobira, Kiyoshi Soyano

**Affiliations:** 1 Institute for East China Sea Research, Organization for Marine Science and Technology, Nagasaki University, 1551-7, Taira-Machi, Nagasaki, 851-2213 Japan; 2 Graduate School of Fisheries and Environmental Sciences, Nagasaki University, 1-14, Bunkyo-Machi, Nagasaki, 852-8521 Japan; 3 Faculty of Fisheries, Nagasaki University, 1-14, Bunkyo-Machi, Nagasaki, 852-8521 Japan; 4 Faculty of Agriculture and Marine Science, Kochi University, 200, Monobe-Otsu, Nankoku, Kochi 783-8502 Japan

**Keywords:** Cephalopod, Reproduction, GnRH-like peptide, Ovary, Testes, Sexual maturation, Gonadal development, Wild squid, Seawater temperature

## Abstract

**Background:**

Cephalopods are widely distributed in oceans worldwide and are important fishery resources. Most species have a lifespan of approximately one year and die after reproduction. In cephalopods, gonadal development may be influenced by seawater temperature; however, the endocrine mechanisms underlying reproductive maturity remain unclear. In recent years, gonadotropin-releasing hormone (GnRH)-like peptide has been identified in invertebrates, including cephalopods, as a possible endocrine regulator, similar to their role in vertebrates. Nevertheless, knowledge of its specific functions in cephalopod reproduction remains limited. This study aimed to clarify the involvement of the endogenous peptide in gonadal development in cephalopods in the bigfin reef squid (*Sepioteuthis lessoniana*). We performed histological observations of gonadal maturation and analyzed brain expression levels and localization of the peptide throughout sexual maturation. Additionally, we examined the relationship between annual gonadal maturation and the seawater temperature cycle.

**Results:**

We identified coding sequences for peptides with conserved functional regions similar to those of other mollusks. Quantitative analysis revealed that brain expression significantly increased during the spermatid stage of testicular development, whereas no association with ovarian development was observed. Immunoreactivity was primarily localized in the optic lobe and around the optic gland, a central site of reproductive regulation in cephalopods. Although ovarian development progressed with increasing seawater temperature, testicular development showed no clear association with the temperature cycle.

**Conclusions:**

These findings suggest that GnRH-like peptides may contribute to early testicular development in *S. lessoniana* through optic gland signaling or direct neural pathways. In contrast, ovarian maturation appears to be strongly influenced by seawater temperature. This study provides foundational insight into the reproductive physiology of cephalopods and highlights regulatory mechanisms governing male and female gonadal development.

**Supplementary Information:**

The online version contains supplementary material available at 10.1186/s40659-025-00626-1.

## Background

Cephalopods are marine animals belonging to the phylum Mollusca, class Cephalopoda [1]. All cephalopod species inhabit marine environments and are widely distributed from coastal regions to the deep sea [2]. With the exception of the genus *Nautilus*, cephalopods are typically short-lived and characterized by rapid growth rates and short life cycles that culminate in a single reproductive season, after which death usually occurs. They exhibit gonochorism, and their breeding seasons vary depending on habitat and species. Many species engage in multiple copulations and spawning events within a single breeding season. Some species reproduce throughout the year, whereas others have more restricted spawning periods. Nevertheless, most cephalopods share a common pattern of maturation with spawning occurring at the end of their life cycle [3].

In vertebrates, the hormonal regulation of gonadal development is governed by the hypothalamus–pituitary–gonad (HPG) axis, involving gonadotropin releasing-hormone (GnRH), gonadotropins (GTHs), and sex steroid hormones [4]. In invertebrates, GnRH-like peptides (GnRH-like) have been identified in at least five major phyla (Cnidaria, Platyhelminthes, Annelida, Mollusca, and Arthropoda) and exhibit functional diversity across taxa [5]. For example, in the bivalve mollusk *Mizuhopecten yessoensis*, administration of synthetic GnRH-like was shown to promote spermatogenesis while inhibiting oocyte development [6]. In contrast, in the gastropod *Aplysia californica*, GnRH-like are unlikely to be directly involved in gonadal stimulation [7].

In cephalopods, GnRH-like have been cloned from several species, including the common octopus (*Octopus vulgaris*), kisslip cuttlefish (*Sepia lycidas*), sword tip squid (*Uroteuthis edulis*), common cuttlefish (*Sepia officinalis*), spotted cuttlefish (*Sepiella japonica*), and pharaoh cuttlefish (*Sepia pharaonic*) [8–12]. While these peptides exhibit low sequence identity and amino acid similarity to vertebrate GnRHs, several conserved functional residues have been noted. For instance, in *O. vulgaris*, structural elements essential for gonadotropin-releasing activity have been identified, including the residue Glu1 at the N-terminus, His4 and Ser6, and the C-terminal Pro11-Gly12-NH_2_ motif [13]. In *O. vulgaris* and spear squid (*Loligo breekeri*), *gnrh-like* expression is primarily localized in central brain regions [14, 15]. Notably, GnRH-like from *O. vulgaris* have been shown to induce luteinizing hormone (LH) release in anterior pituitary cells of *Coturnix japonica* (quail) in vitro, suggesting a conserved mechanism of gonadotropin regulation [13]. Furthermore, GnRH-like stimulate steroidogenesis, specifically, the production of testosterone, progesterone, and 17β-estradiol, in both ovaries and testes of *O. vulgaris*, where GnRH-like receptors are abundantly expressed. These findings suggest that GnRH-like may exert local effects by activating their own receptors within the gonads and influencing steroid hormone synthesis [8]. These results suggest the presence of short GnRH-like endocrine loops in cephalopod gonads, and it is hypothesized that gonads are not only targets of hormones but also sites of hormone synthesis.

Previous studies on the functional role of GnRH-like in the cephalopod cuttlefish *S. japonica* revealed that the vitellogenin gene (*Vg1*) showed higher expression localization in the ovary and liver than in the other tissues, and the additional in vitro experiment of GnRH-like in the ovarian and liver tissues significantly increased the *Vg1* expression levels in each tissue in a concentration-dependent manner, suggesting the involvement of GnRH-like on the vitellogenesis [16]. Additionally, we recently reported that *gnrh-like* was highly expressed prior to gonadal sex differentiation in the cuttlefish *Sepia lycidas*; however, no sex-specific differences in expression were observed throughout early ontogenesis. This suggests that GnRH-like may not be directly involved in gonadal differentiation, but rather may play a role in early ontogenesis and juvenile growth [9, 17]. These findings indicate that GnRH-like are broadly distributed among cephalopods. However, information on their functional role in reproductive regulation remains limited.

The bigfin reef squid, *Sepioteuthis lessoniana*, belongs to the family Loliginidae and inhabits coral reefs, rocky areas, and sea grass or seaweed beds. Due to its broad distribution across the Indo-Pacific region, *S. lessoniana* is an economically important species in many countries [18]. Consequently, there is a growing interest in developing aquaculture techniques for this species. Despite its importance, endocrine regulation of sexual maturation, spawning, and spermiogenesis remains poorly understood in this species. Notably, there are no reports involving wild individuals or detailed histological observations of gonads from *S. lessoniana* collected from coastal environments. Furthermore, the environmental factors that induce gonad development in this species are unknown. While vitellogenin expression has been measured in the ovary, *gnrh-like* expression during sexual maturation, particularly its potential role as an upstream regulator of vitellogenesis, has not been examined [18]. It is widely accepted that seawater temperature is one of the most influential environmental factors affecting gonadal development, hatching, and ontogenesis in cephalopods [19, 20]. However, the relationship between seawater temperature and the timing of sexual maturation in *S. lessoniana* remains uncharacterized.

In this study, we aimed to clarify the annual gonadal developmental process in *S. lessoniana* and investigate the roles of endogenous GnRH-like and seawater temperature in gonadal development. Histological observations of gonads, molecular cloning and sequencing of *gnrh-like*, analysis of its expression in the brain, and monitoring of seawater temperature fluctuations were conducted to provide new insights into the endocrine regulation of reproduction in cephalopods.

## Methods

### Ethical use of animals

This study was approved by the Animal Care and Use Committee of the Faculty of Fisheries at Nagasaki University (approval no. NF-0063), in accordance with the Guidelines for Animal Experimentation of the Faculty of Fisheries (for fish, amphibians, and invertebrates) and institutional animal care regulations.

### Animals and sampling procedures

In this study, brain tissue from dead squid was not used for expression analysis due to potential RNA degradation. Wild *S. lessoniana* were collected from coastal waters near Nagasaki, Japan, between 2018 and 2023, either by line fishing using the training vessel “Kakuyo-maru,” or by purchasing freshly caught individuals from local fish markets. Live specimens were anesthetized in 1% ethanol-seawater, following the procedure outlined by Ikeda et al. (2009) [21]. Body weight, dorsal mantle length, gonadal weight, and reproductive appendage weight were recorded. The mantle was ventrally incised to remove the gonads and brain. Excised gonads and brains were fixed in Davidson's solution at room temperature (15–25 °C) for 4 days, then transferred to 70% ethanol and stored at 4 °C until histological analysis. For quantification of *gnrh-like* expression, brain tissues were preserved in RNAlater Solution (Thermo Fisher Scientific, Waltham, MA, USA) at 4 °C overnight and stored at − 30 °C until RNA extraction.

### Surface seawater temperature measurements around Nagasaki

To assess the correlation between gonadal development and environmental temperature, seawater temperature was obtained on an aquaculture test raft maintained by the Nagasaki Prefectural Institute of Fisheries (Taira-machi, Nagasaki City, Nagasaki Prefecture) from a depth of 5 m. Weekly seawater temperature measurements were accessed from the publicly available monitoring system (https://www.pref.nagasaki.jp/bunrui/shigoto-sangyo/suisangho/suisan-shiken-suishi-teichi-water-temperature/). Data collected between September 1, 2021, and June 29, 2022 were representative of seasonal temperature changes during the study period.

### Calculation of gonadosomatic index (GSI)

The gonadosomatic index (GSI) was calculated using the following equation:

GSI (%) = (gonad weight/body weight) × 100.

### Histological observation of gonads

Fixed gonads stored in 70% ethanol were dehydrated, permeabilized using an ethanol–Lemosol series, and embedded in paraffin following standard protocols. Tissue Sections. (5 μm thick) were prepared using a rotary microtome (HM325; Thermo Fisher Scientific) and stained with Carrazzi’s hematoxylin and 1% eosin. Sections were examined using an optical microscope (Axioscope 5; Carl Zeiss AG, Oberkochen, Germany). Maturity staging was performed based on the gonadal developmental stages described for *U. edulis* [22].

### Molecular cloning of *gnrh-like*

Total RNA was extracted from brain tissue using ISOGEN II (Nippon Gene, Tokyo, Japan) in accordance with the manufacturer’s protocol. RNA concentrations were measured using a NanoDrop 2000 spectrophotometer (Thermo Fisher Scientific), and samples were stored at − 80 °C until cDNA synthesis.

Three micrograms of total RNA were reverse transcribed into cDNA using oligo (dT) primers and the PrimeScript II 1 st strand cDNA Synthesis Kit (Takara Bio Inc., Shiga, Japan), following the manufacturer's instructions. Synthesized cDNA was stored at − 30 °C until use in PCR. PCR amplification of the *gnrh-like* gene was performed using degenerate primers designed for cephalopods, as described in our previous study [9], and Tks Gflex DNA Polymerase (Takara Bio Inc.) according to the manufacturer’s protocol.

PCR products were separated via 2.0% TBE agarose gel electrophoresis and purified from excised gel fragments using the QIAquick Gel Extraction Kit (Qiagen, Hilden, Germany). The purified product was ligated into a plasmid vector using the TOPO TA Cloning Kit (Invitrogen) after adding 3ʹ A-overhangs. Recombinant plasmids were transformed into *Escherichia coli* strain DH5α (Competent Quick DH5α; Toyobo, Osaka, Japan). Blue-white colony screening was used to identify transformants containing inserts. Plasmid DNA was extracted from positive clones using the NucleoSpin Plasmid EasyPure kit (Marcherey-Nagel, Düren, Germany) and sequenced using FASMAC (Atsugi, Japan).

Based on the preliminary *gnrh-like* sequence obtained, rapid amplification of cDNA ends (RACE) was performed to isolate the full-length 5′ and 3′ ends using the SMARTer RACE 5′/3′ kit (Takara Bio Inc.) according to the manufacturer’s instructions. mRNA for RACE was purified from total RNA using the Oligotex-dT30 < Super > mRNA Purification Kit (Takara Bio Inc.). RACE PCR was conducted using PrimeSTAR Max DNA Polymerase (Takara Bio Inc.) with gene-specific primers (GSPs) and a Universal Primer A Mix. The primers used included GSPs based on the vector sequence for the 5′-RACE (5′-gattacgccaagcttcttcgtttaccgccagggtgccatc) and 3′-RACE (5′-gattacgccaagcttgcaccctggcggtaaacgaagtggaa).

### Alignment

To confirm sequence homology and identity, the obtained *gnrh-like* sequences were aligned with known GnRH-like genes sequences from other species. Sequences were analyzed using BLAST and subjected to multiple sequence comparison using the log-expectation tool (MUSCLE) for cross-species alignment [23, 24].

### Quantification of *gnrh-like* expression levels

#### RNA extraction

Only live individuals were used for brain GnRH-like gene expression analysis due to the requirement for high RNA integrity. Total RNA was extracted following the protocol described previously. RNA concentration was measured using a NanoDrop 2000 spectrophotometer and diluted to 100 ng/µL with RNase free water. RNA samples were stored at − 80 °C until cDNA synthesis.

### cDNA synthesis

For each sample, 500 ng of total RNA was reverse transcribed in a 10 µL reaction volume using the ReverTra Ace qPCR RT Master Mix with gDNA Remover (Toyobo), following the manufacturer’s instructions. The resulting cDNA was diluted five-fold using UltraPure Distilled Water (Thermo Fisher Scientific) and stored at − 30 °C until quantitative real-time PCR (qPCR).

### qPCR

qPCR was performed to quantify *gnrh-like* expression in the brains of *S. lessoniana*. To generate a standard curve, the *gnrh-like* gene was recloned using primers designed with Primer3Plus (forward: 5′-cagacaactgccaaccaaga, reverse: 5′-gttggtgcaggtggtgagagagta). Amplicons were ligated, propagated, purified, and sequenced as described earlier. A 268 bp partial sequence was confirmed and used as the plasmid standard for estimating *gnrh-like* copy numbers. For qPCR, a primer pair specific to the target gene was designed using Primer3Plus (forward: 5′-actatcctcctcctgtctttgtg, reverse: 5′-gctttcgtctggggtctg). PCR was performed in a 10 µL reaction containing 10 ng total RNA equivalent of diluted cDNA, 2 × KAPA SYBR FAST qPCR Master Mix (Kapa Biosystems, Wilmington, MA, USA), and 100 nM of each primer. The amplification protocol included an initial enzyme activation step at 95 °C for 3 min, followed by 40 cycles of 95 °C for 3 s and 60 °C for 30 s (denaturation, annealing, extension). Reactions were held at 4 °C after cycling, and melting curve analysis was performed to confirm specificity and detect any nonspecific amplification. PCR amplification and fluorescence detection were performed using the StepOnePlus software (Thermo Fisher Scientific).

### Immunohistochemistry of GnRH-like in the brain

A synthetic oligopeptide corresponding to a partial GnRH-like amino acid sequence (CNGWHPGGKRSGIPDM) was used to generate a specific antibody. Specific pathogen-free Japanese white rabbits were immunized four times at two-week intervals with the synthesized oligopeptide by Cosmo Bio (Tokyo, Japan).

Brain samples fixed in Davidson’s solution were used for immunohistochemistry (IHC). The method used to investigate GnRH-like expression in the brain of *S. lessoniana* was adapted from Murata et al. (2021) [9], with minor modifications. Briefly, we first made 5 μm-thick cross-sections of the brain samples. Then, the representative parts of the paraffin-embedded brain sections were deparaffinized with xylene, rehydrated through a graded ethanol series, and rinsed in phosphate-buffered saline. Endogenous peroxidase activity was blocked by incubating the sections in 3% H_2_O_2_/methanol for 15 min. Nonspecific binding was reduced by pre-incubation with 10% normal goat serum for 15 min. Sections were then incubated overnight at 4 °C in a moisture chamber with the primary antibody (anti-GnRH-like) diluted 1:2,000 in Primary Antibody Diluent (Sakura Finetek USA, Inc., Torrance, CA, USA). Immunoreactivity was detected using the Histidine anti-rabbit IHC kit (Nichirei, Tokyo, Japan) and visualized using diaminobenzidine as the chromogen. Brain sections visualizing the immunoreactivity against GnRH-like were examined using light microscopy (APEXVIEW APX100, Evident Corp., Tokyo, Japan). Immunoreactivity distribution was observed and compared between three male and three female *S. lessoniana* individuals collected during the breeding season (April to June) and the non-breeding season (September to November), thus a total of 12 individuals were used for IHC. Brain regions examined for IHC were determined as described by Amano et al. [15].

﻿To test the specificity of the immunoreactions, adjacent sections served as controls and were treated under the following conditions: primary antibody pre-absorbed with a 50-fold excess of the original antigen (diluted 1:2,000 in Primary Antibody Diluent) in accordance with the past reports [9, 15], Primary Antibody Diluent alone, and pre-immune serum from the rabbit used for antibody production (diluted 1:2,000 in Primary Antibody Diluent). We used the sections showing the immunoreactivity against GnRH-like of the brains from the representative four individuals for the control sections.

### Statistical analysis

Multiple comparisons were conducted using the Tukey–Kramer test for GSI among each month, as the data was confirmed to be normally distributed using the Shapiro–Wilk test. On the other hand, the Steel–Dwass method was adopted to assess differences in brain *gnrh-like* expression levels in individuals of each gonadal maturation stage, after the data was not confirmed to be normally distributed using the Shapiro–Wilk test. In the comparison of brain *gnrh-like* expression levels, the late yolkless stage in females was excluded from the analysis due to the small sample size (n < 3). All statistical analyses were conducted using R version 4.1.3 (R Foundation for Statistical Computing, Vienna, Austria) and RStudio 2023.03.0 + 386 (Posit, Boston, MA, USA).

## Results

### Circumannual changes in GSI and seawater temperature around Nagasaki

**Fig. 1:**
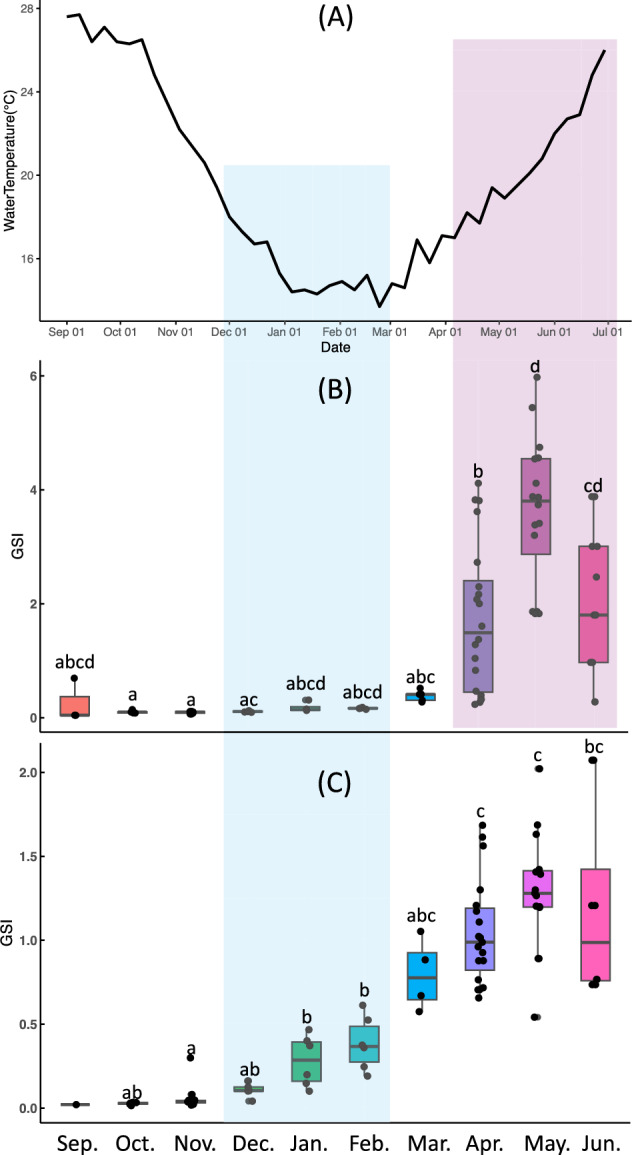
Annual gonadal development of *Sepioteuthis lessoniana* and seawater temperature change. Monthly changes in seawater temperature around Nagasaki (**A**) and gonadosomatic index (GSI) in female (**B**) and male (**C**) *S. lessoniana*. Different letters in (**B**) and (**C**) indicate statistically significant differences (*α* < 0.05). Red and blue shaded areas indicate the correlation of ovarian and testicular maturation, respectively, with water changes

Seawater temperature data collected from the coastal waters of Nagasaki City during the experimental period are shown in Fig. 1 A. The highest recorded temperature was 27.7 °C on September 8, which subsequently declined to a seasonal low of 13.7 °C on February 23. From March onward, seawater temperature steadily increased.

We compared seasonal changes in the GSI between male and female *S. lessoniana* individuals (Fig. 1 B and C). In females, the GSI remained relatively constant from September to December and gradually increased from January to March. A more rapid increase was observed beginning in April, with a significant rise from March to May consistent with the increase in water temperature (Fig. 1 A and B). In males, the GSI remained largely unchanged between September and March, although a gradual increase was noted beginning in December before the water temperatures start to rise (Fig. 1 A and C). A significant increase occurred between February and April (Fig. 1 C). After March, no further significant changes in male GSI were observed (Fig. 1 C). Notably, the GSI of mature females in May was more than twice that of males (Fig. 1 B and C).

### Developmental stages of gonads based on histological characteristics and seasonal appearance of each stage in wild *S. lessoniana*

**Fig. 2:**
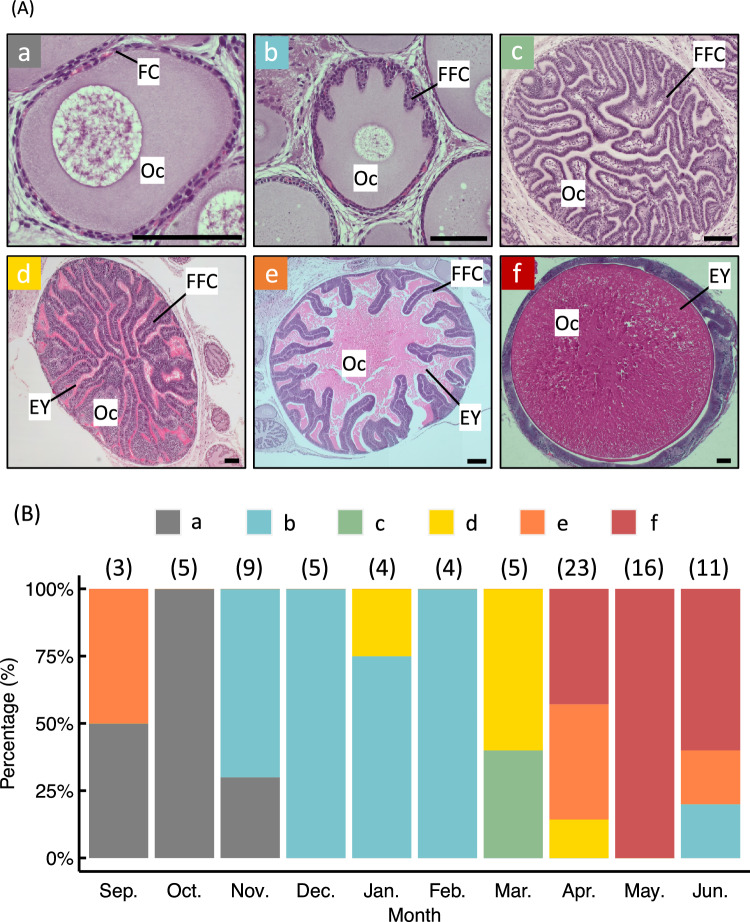
Histological images and monthly appearance of each ovarian maturation stage in *Sepioteuthis lessoniana*. (**A**) Ovarian sections of *S. lessoniana*. Based on histological characteristics, oocyte development was divided into six stages: cytoplasmic growth (**a**), early yolkless (**b**), late yolkless (**c**), early vitellogenic (**d**), late vitellogenic **(e)**, and ripe (**f**). Oc, oocyte; FC, follicular cell; FFC, follicular-fold complex; EY, egg yolk. Scale bars = 100 μm. (B) Monthly distribution of female maturation stages: grey, cytoplasmic growth; blue, early yolkless; green, late yolkless; yellow, early vitellogenic; orange, late vitellogenic; red, ripe stage. The numbers in brackets above each bar indicate the sample sizes of each month

In this study, we collected 170 wild *S. lessoniana* specimens, including 57 live and 113 dead individuals, from September to June during the years 2018 and 2021 to 2023. Female gonadal maturation was classified based on the most advanced oocyte stage observed in the ovary. Oocyte development was divided into six stages according to histological characteristics as follows: cytoplasmic growth stage, in which follicular cells surround the oocyte (Fig. 2 A – a); early yolkless stage, when follicular cells begin forming the follicular-fold complex (FFC) within the oocyte (Fig. 2 A – b); late yolkless stage, when the FFC is present but eosin-stained yolk material is absent (Fig. 2 A – c); early vitellogenic stage, characterized by a completed FFC and the active accumulation of eosinophilic yolk material (Fig. 2 A – d); late vitellogenic stage, in which yolk accumulates and the FFC is displaced toward the periphery of the oocyte (Fig. 2 A – e); and the ripe stage, defined by complete migration of follicular cells around the oocyte, indicating readiness for ovulation (Fig. 2 A – f).

The annual occurrence of each maturation stage in females is shown in Fig. 2 B. Both late vitellogenic and cytoplasmic growth stage individuals were observed in September, while only cytoplasmic growth stage individuals were recorded in October. Individuals in the early yolkless stage first appeared in November. By March, individuals in the late yolkless and early vitellogenic stages were present. Ripe-stage individuals were first observed in April. In May, only ripe-stage individuals were found, while in June, both immature and ripe stage individuals were detected.

**Fig. 3:**
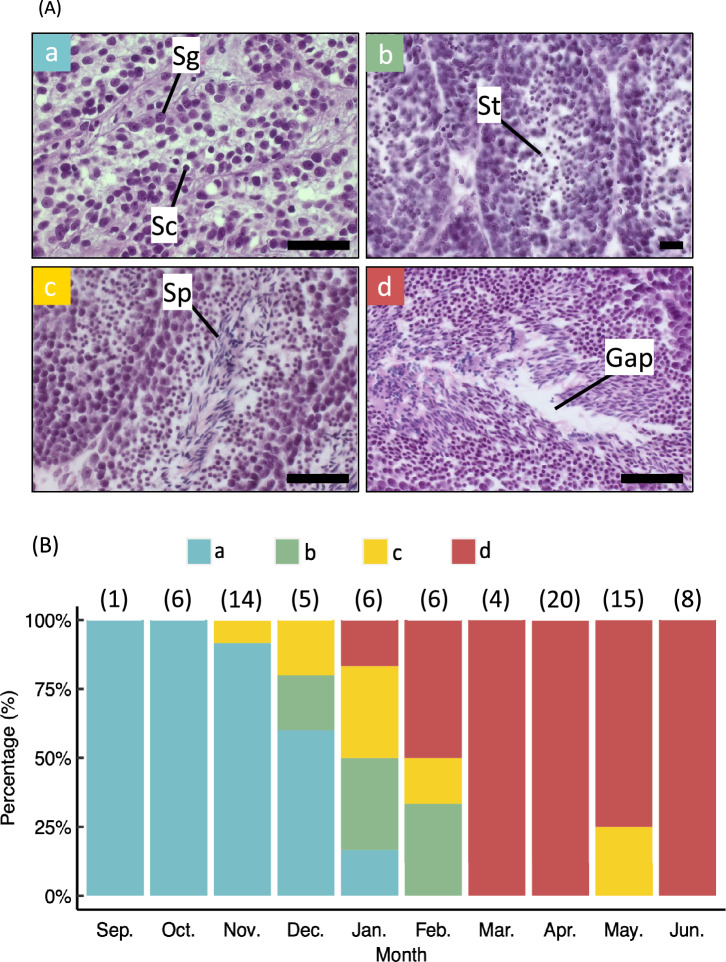
Histological images and monthly appearance of each testicular maturation stage in *Sepioteuthis lessoniana*. **A** Testicular sections of *S. lessoniana*. Based on histological characteristics, testicular development was divided into four stages: spermatocyte (**a**), spermatid (**b**), late spermatogenesis (**c**), mature (**d**); Sg, spermatogonia; Sc, spermatocyte; St, spermatid; Sp, sperm; Gap, created by prolific ejaculation. Scale bars = 50 μm. **B** Monthly distribution of male maturation stages: blue, spermatocyte; green, spermatid; orange, late spermatogenesis; red, mature stage. The numbers in brackets above each bar indicate the sample sizes of each month

Male gonadal development was categorized into four stages based on histological features: spermatocyte stage (GSI = 0.042 ± 0.006 SE), in which both spermatogonia and spermatocytes are observed in the seminiferous lobules but no spermatids or sperm are present (Fig. 3 A – a); spermatid stage (GSI = 0.157 ± 0.047 SE), where spermatids are visible in the lobules, but sperm are still absent (Fig. 3 A – b); late spermatogenesis stage (GSI = 0.778 ± 0.114 SE), marked by active spermatogenesis and the appearance of sperm within the lobules (Fig. 3 A – c); and mature stage (GSI = 1.119 ± 0.068 SE) characterized by abundant sperm and visible interspaces, indicating ejaculation (Fig. 3 A – d).

The seasonal distribution of each testicular development stage is shown in Fig. 3 B. Only spermatocyte stage individuals were observed in September and October. Spermatid-stage individuals appeared in November, followed by late spermatogenesis-stage individuals in December. Mature individuals were first recorded in January, and by March, all sampled males had reached the mature stage.

### Cloning of *gnrh-like* from *S. lessoniana*

RACE using gene-specific primers yielded ﻿overlapping 5′ and 3′ sequences, resulting in a full-length of 730 bp (GenBank accession no. LC866554). The nucleotide sequence was compared with those of other cephalopods: *U. edulis* (AB447557), *S. japonica* (KP982885), *S. lycidas* (LC550284), and *S. pharaonis* (MY211953). Sequence identities were 87, 82, 81, and 82%, respectively, and 76%, with an additional unlisted species (Additional file 1 and 2 A). The translated amino acid sequence consisted of 89 residues. When aligned with GnRH-like amino acid sequences from *U. edulis* (BAH09303.1), *S. japonica* (ALS92801.1), *S. lycidas* (BCG29712.1), *S. pharaonis* (QPB69198.1), and *O. vulgaris* (BAB86782.1), sequence identities were 92, 87, 83, 82, and 69%, respectively. Including amino acids with similar functional properties, the overall similarity reached 94, 90, 87, 86, and 80%, respectively (Additional file 2 A). Comparative analysis of functional regions in GnRH-like revealed high conservation among cephalopod species. However, partial differences were observed in *M. yessoensis* (phylum Bivalves) and *A. californica* (phylum Gastropoda), suggesting evolutionary divergence in molluscan lineages (Additional file 2 B).

### Quantification of *gnrh-like* expression in the brain during gonadal maturation

**Fig. 4:**
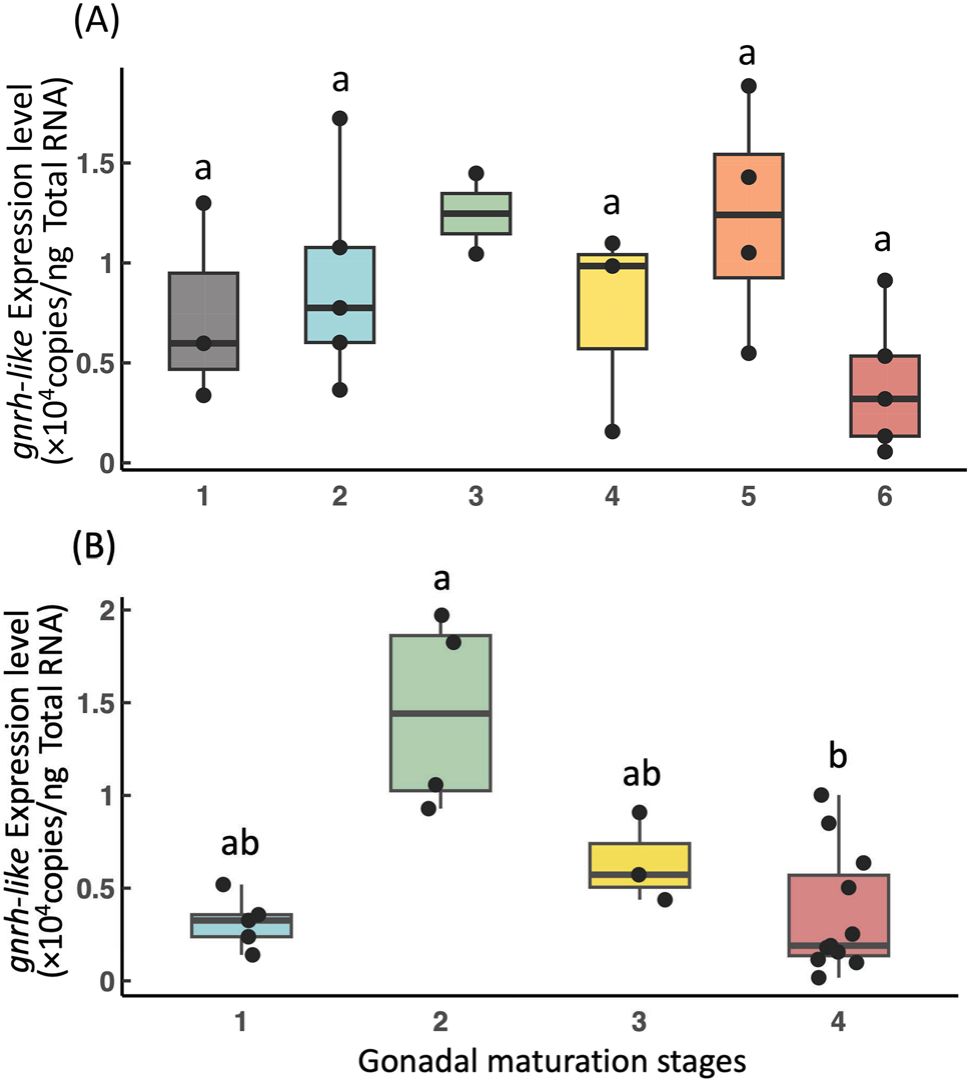
GnRH-like gene (*gnrh-like*) expression profiles during gonadal development in *Sepioteuthis lessoniana*. *gnrh-like* expression levels in the brain at different gonadal maturation stages in female (**A**) and male (**B**) *S. lessoniana*. (**A**): grey, cytoplasmic growth stage (1) ; blue, early yolkless (2) ; green, late yolkless (3) ; yellow, early vitellogenic (4); orange, late vitellogenic (5); red, ripe (6) . (**B**): blue, spermatocyte stage (1) ; green, spermatid (2) ; yellow, late spermatogenesis (3) ; red, mature (4) . Different letters indicate statistically significant differences (*α* < 0.05) except for stage 3 in female due to the small sample size

In females, *gnrh-like* expression levels in the brain remained uniform across all stages of gonadal development, with no statistically significant differences observed except for stage 3 due to the small sample size (Fig. 4 A). In contrast, male individuals exhibited higher expression levels at the spermatid stage compared to other stages. A statistically significant difference was detected between the spermatid and mature stages (Fig. 4 B).

### Immunohistochemical distribution of GnRH-like in the brain

**Fig. 5:**
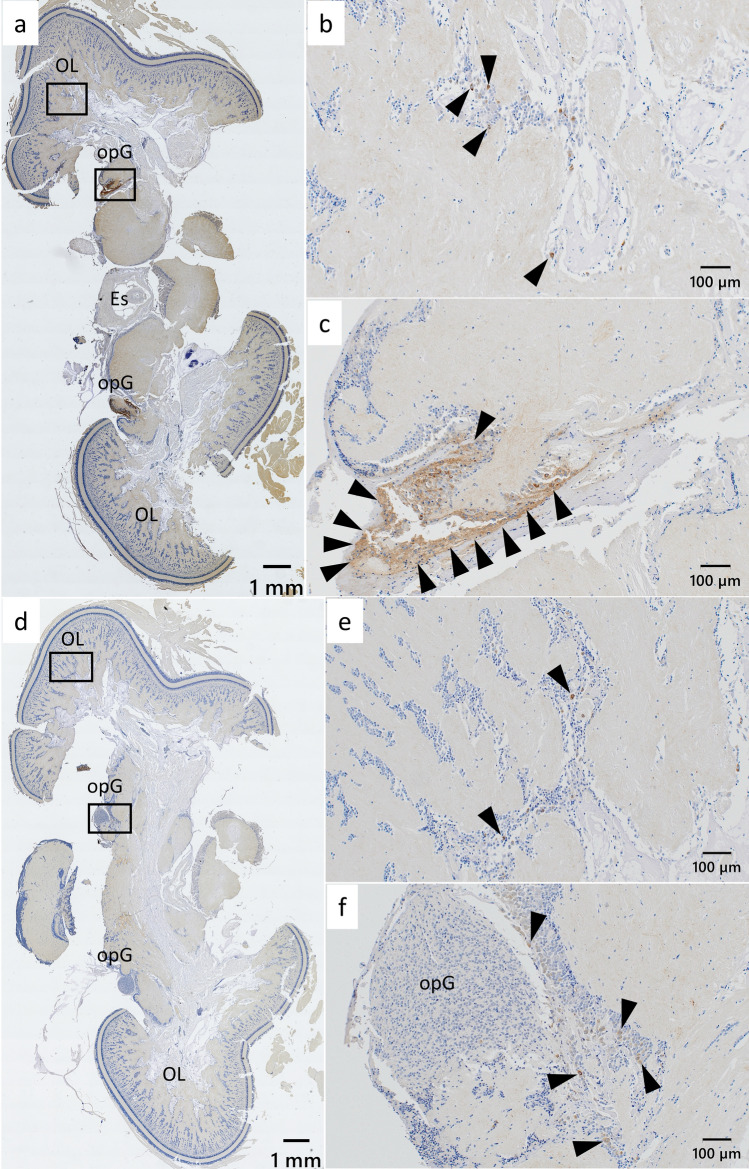
Immunohistochemical distribution of GnRH-like peptide in the brain of immature female *Sepioteuthis lessoniana*. Low-magnification images of transverse brain sections at anterior (**a**) and middle (**d**) levels. High-magnification views of the optic lobe (**b** and **e**) and optic gland (**c** and **f**) correspond to areas marked by black squares in (**a**) and (**d**). Black arrowheads indicate representative GnRH-like immunoreactive signals. ES, esophagus; OL, optic lobe; opG, optic gland. Scale bars = 1 mm (**a** and **d**) and 100 µm (**b**, **c**, **e**, and **f**)

Immunoreactivity against GnRH-like was observed in neural cell bodies and fibers predominantly distributed around the optic lobe and in the region adjacent to the optic gland (Fig. 5 a and d). Immunoreactive cell bodies were scattered throughout the optic lobe (Fig. 5 b and e), and were localized near the optic gland; however, no immunoreactive cell bodies were detected within the main structure of the optic gland itself (Fig. 5 c and f). No differences in the distribution or intensity of GnRH-like immunoreactivity were observed between sexes or across seasons using three immature males, three immature females, three mature males, and three immature females. In control sections, no significant background immunoreactivity or staining intensity difference was detected in any of the experimental conditions (Additional file 3).

## Discussion

In this study, the annual gonadal developmental cycle was characterized histologically, and the GSI was calculated. In females, the GSI increased rapidly from March to May, with some individuals reaching final maturity in April. In contrast, the GSI in males increased gradually from November, reaching final maturity in January, ahead of females. These results indicate that testicular development proceeds more rapidly than ovarian development in the reproductive cycle of *S. lessoniana*, which is consistent with a previous report describing the gonadal cycle of another cephalopod species [25]. These findings also support previous suggestions that the completion of testicular development prior to ovarian development may be a common feature among cephalopods [25]. Based on the observed variations in gonadal development and GSI, the spawning season of *S. lessoniana* in the coastal waters of Nagasaki City was estimated to begin in April, with peak spawning likely occurring in May and June.

Seawater temperature is a critical environmental factor influencing sexual maturation and spawning in marine organisms [26]. Previous studies have indicated that cephalopods have a suitable seawater temperature range for spawning and that increasing temperatures can act as a trigger for final maturation [19, 20]. Our results revealed that in males, sexual maturation progressed as seawater temperature decreased, with males reaching final maturity in January, when the water temperature was at its annual minimum of 14 °C. This finding suggests that testicular development in *S. lessoninana* may proceed independently of temperature increases. In contrast, females entered the early vitellogenic stage between February and March, coinciding with the seasonal rise in seawater temperature, indicating that increasing temperature may serve as a cue for the onset of yolk accumulation. Previous studies have reported that juvenile *S. lessoniana* require seawater temperatures of 20 °C or higher for normal development [18]. In the coastal waters of Nagasaki, seawater temperature reached 17.0 °C in April and 20.1 °C in May. Therefore, it is plausible that females that reached reproductive readiness after April spawned during May, coinciding with temperatures conducive to juvenile development.

This study represents the first successful cloning of *gnrh-like* in *S. lessoniana*. The cloned sequences showed high similarity to those of other mollusks, particularly *U. edulis* [10], and shared conserved functional regions, suggesting a common ancestral origin. Furthermore, we demonstrated an association between gonadal maturation and brain *gnrh-like* expression in *S. lessoniana*. Expression levels of *gnrh-like* in the brain showed an increasing trend during the spermatid stage of testicular development, and was significantly higher at the spermatid stage than at the mature stage. These facts suggest that GnRH-like may be involved in early spermatogenesis in *S. lessoniana*. Similar findings have been reported in the scallop *M. yessoensis,* where administration of synthetic GnRH-like accelerated spermatogenesis [6], further supporting the possible role for GnRH-like in testicular development in *S. lessoniana*. Additionally, immunohistochemical analysis showed that *gnrh-like* expression was localized primarily around the optic gland, a region long hypothesized to play a central role in cephalopod reproduction regulation [27, 28]. The results of our control experiment conducted in accordance with the past reports clearly demonstrate the immunoreactive specificity of the antibody used in this study [9, 15]. Based on these findings, we suggest that GnRH-like may be involved in early testicular development via actions mediated through, or near, the optic gland in *S. lessonianna*. Future studies should focus on both in vivo and in vitro analyses of synthetic GnRH-like peptide function in male testes.

However, we observed no significant relationship between brain *gnrh-like* expression and ovarian maturation in *S. lessoniana*. To elucidate the involvement of the GnRH-like peptide in ovarian development, we will need to investigate the receptor expression profile and perform functional analysis using peptides in vitro as reported in Cephalopod cuttlefish, *S. japonicus* [16]. These studies may also provide fundamental information for the potential of the peptide in inducing gonadal maturation. Such investigations will enhance our ability to sustainably manage wild populations and support aquaculture expansion for *S. lessoniana* and related cephalopod species.

## Conclusion

This study demonstrated that seawater temperature may play a role in ovarian development, whereas testicular development in *S. lessoniana* appears to proceed with no apparent association of seasonal seawater temperature fluctuations. We also characterized the expression patterns and brain localization of GnRH-like proteins in relation to gonadal development, suggesting that these proteins may function during early testicular development via the optic gland or adjacent regions in *S. lessoniana.* These findings significantly advance our understanding of reproductive endocrinology in cephalopods, particularly for economically important species like *S. lessoniana*.

## Supplementary Information

Additional file 1. Comparison of *gnrh-like* nucleotide sequences between *Sepioteuthis lessoniana* and other cephalopods: *Uroteuthis edulis*, *Sepiella japonica*, *Sepia Lycidas*, *Sepia pharaonis*, and *Octopus vulgaris*. Asterisks indicate conserved nucleotides. Red and green boxes denote primer regions used for primary PCR and qPCR, respectively. Orange highlights indicate partial *gnrh-like* sequences cloned in this study. Additional file 2. (A) Identity and similarity of GnRH-like peptide nucleotide and amino acid sequences among *Sepioteuthis lessoniana* and other cephalopods: *Uroteuthis edulis*, *Sepiella japonica*, *Sepia lycidas*, *Sepia pharaonis*, and *Octopus vulgaris*. (B) Conservation of functional GnRH-like regions among cephalopods and other mollusks: scallop *Mizuhopecten yessoensis* and sea slug *Aplysia californica*. Asterisks indicate identical amino acids. Additional file 3. Adjacent brain sections of *Sepioteuthis lessoniana* immunostained with: anti-GnRH-like (a), antigen-absorbed anti-GnRH-like antibody (b), Primary Antibody Diluent only (c), and pre-immune serum (d). Arrowheads in (a) indicate representative GnRH-like immunoreactivity. Scale bars = 100 µm.

## Data Availability

All data generated or analyzed during this study are included in this published article.

## Abbreviations

GnRH: Gonadotropin releasing-hormone
GTHs: Gonadotropins
HPG: Hypothalamus–pituitary–gonad
GnRH-like: GnRH-like peptides
GSI: Gonadosomatic index
FFC: Follicular-fold complex
