# Correction: Relationship between seawater temperature, brain GnRH-like peptide expression, and gonadal development in wild bigfin reef squid (*Sepioteuthis lessoniana*)

**DOI:** 10.1186/s40659-025-00632-3

**Published:** 2025-08-02

**Authors:** Umina Kubo, Jaewoo Lee, Ryosuke Murata, Takashi Aoshima, Yuji Mushirobira, Kiyoshi Soyano

**Affiliations:** 1 Institute for East China Sea Research, Organization for Marine Science and Technology, Nagasaki University, 1551-7, Taira-Machi, Nagasaki, 851-2213 Japan; 2 Graduate School of Fisheries and Environmental Sciences, Nagasaki University, 1-14, Bunkyo-Machi, Nagasaki, 852-8521 Japan; 3 Faculty of Fisheries, Nagasaki University, 1-14, Bunkyo-Machi, Nagasaki, 852-8521 Japan; 4 Faculty of Agriculture and Marine Science, Kochi University, 200, Monobe-Otsu, Nankoku, Kochi 783-8502 Japan

**Correction: Biological Research (2025) 58:46**

10.1186/s40659-025-00626-1

In this article the author’s name Jaewoo Lee was incorrectly written as Lee Jaewoo.

The original article has been corrected.

