# Supplementary material for: Relationship between seawater temperature, brain GnRH-like peptide expression, and gonadal development in wild bigfin reef squid (Sepioteuthis lessoniana)
Source: Biol Res. 2025 Jul 2;58:46. doi: 10.1186/s40659-025-00626-1 (PMC12225108; doi:10.1186/s40659-025-00626-1)
Supplement: Supplementary file 1 — Additional file 1. Comparison of gnrh-like nucleotide sequences between Sepioteuthis lessoniana and other cephalopods: Uroteuthis edulis, Sepiella japonica, Sepia Lycidas, Sepia pharaonis, and Octopus vulgaris. Asterisks indicate conserved nucleotides. Red and green boxes denote primer regions used for primary PCR and qPCR, respectively. Orange highlights indicate partial gnrh-like sequences cloned in this study. Additional file 2. (A) Identity and similarity of GnRH-like peptide nucleotide and amino acid sequences among Sepioteuthis lessoniana and other cephalopods: Uroteuthis edulis, Sepiella japonica, Sepia lycidas, Sepia pharaonis, and Octopus vulgaris. (B) Conservation of functional GnRH-like regions among cephalopods and other mollusks: scallop Mizuhopecten yessoensis and sea slug Aplysia californica. Asterisks indicate identical amino acids. Additional file 3. Adjacent brain sections of Sepioteuthis lessoniana immunostained with: anti-GnRH-like (a), antigen-absorbed anti-GnRH-like antibody (b), Primary Antibody Diluent only (c), and pre-immune serum (d). Arrowheads in (a) indicate representative GnRH-like immunoreactivity. Scale bars = 100 µm. [file 40659_2025_626_MOESM1_ESM.pdf]

```
S. japonica     ------------------------------------------------------------
S. lycidas      ------------------------------ACATGGGC----------------------
S. pharaonis    ------------------------------------------------------------
S. lessoniana   ATAGGGCAAGCAGTGGTATCAACGCAGAGTACATGGGGAGTGATCTCCCCTATTTAAGCG
U. edulis       ------------------------------------------------------------

S. japonica     ----------------------ACTTACTTTTCTCTCATCCCTCGAAGAAGAC-AAAGAA
S. lycidas      ----------------------ACTTCCTTTACACTCGTCCCTCGAAGAAGACAAAAGAA
S. pharaonis    ----------------------ACTTCCTTTACACTCGTCCCTCGAAGAAGACAAAAGAA
S. lessoniana   AAGATCTCATAGAAATTCCTTCACTTTCCCTTCTCTCATCTCTTGAAG-------ACAAA
U. edulis       ------------------------------------CATCTTTTGAAG-------ACAAA
                                                    * **  * ****       *  **

S. japonica     CAGTTGAAATCTCCATCATCAGG--TAAACATCTACCAGCCAGTATAATCATGTCAACCA
S. lycidas      CACTTCAAATCTCCATCATCAGC--TAAACATCTACCAGACAGTATTATCATGTCAACCT
S. pharaonis    CACTTCAAATCTCCATCATCAGC--TAAACATCTACCAGACAGTATTATCATGTCAACCT
S. lessoniana   CTCTCCAAG-CTTAATCGTCAGAGTCAGACAACTGCCAACCAAGAGTATCATGTCAACCT
U. edulis       CACCGCAAAGCTTAATCGTCAGA--CAACTATTT-CCAGCCAG---CATCATGTCAACTT
                *     **  **  *** ****    *   *  * ***   **     ***********

S. japonica     CTGCACTCTCGTCCAACTTGAGAAAAATGGCCTTTCTCACCTGTGCCATTCTCCTCCTGT
S. lycidas      CCACAGCCTCGTCCAGCCTGAGAAGAATGGCCTTTTTCACCTGTGCTATTCTTCTCCTCT
S. pharaonis    CCACAGCCTCGTCCAGCCTGAGAAGAATGGCCTTTTTCACCTGTGCTATTCTTCCCCTCT
S. lessoniana   GCCCAGTCTCTTCCACTCTAAGGAAAATGGTTTTCCTGACCTGCACTATCCTCCTCCTGT
U. edulis       CCCCAGTCACTTCCACTCTCAGAAGAATGGTCTTCCTGACCTGCGCCATCTTCCTCCTGT
                   **  * * ****   * ** * *****  **  * *****  * **  * * *** *

S. japonica     CTTTCTGCATGCAAATACAAGCACAGAATTACCATTTTAGCAATGGATGGCACCCTGGTG
S. lycidas      CTTTCTGCATGCATATCCAGGCACAGAATTACCATTTTAGCAATGGATGGCACCCTGGTG
S. pharaonis    CTTTCTGCATGCATATCCAGGCACAGAATTACCATTTTAGCAATGGATGGCACCCCGGTG
S. lessoniana   CTTTGTGCATGCAGACACAGGCACAGAATTACCATTTTAGCAATGGATGGCACCCTGGCG
U. edulis       CTTTGTGCATGCAGACACAGGCACAGAATTACCATTTCAGCAACGGATGGCACCCTGGCG
                **** ******** *  ** ***************** ***** *********** ** *

S. japonica     GTAAACGAAGTGGACTTCCAGACATGCAGTGTCATTTCAGACCCCAAACAAAAGCTTTGA
S. lycidas      GTAAACGAAGTGGACTTCCAGACATGCAGTGTCATTTCAGACCACAAACAAAAGCTTTGA
S. pharaonis    GTAAACGAAGTGGACTTCCAGACATGCAGTGTCATTTCAGACCACAAACAAAAGCTTTGA
S. lessoniana   GTAAACGAAGTGGAATTCCAGACATGCAGTGTCGTTTCAGACCCCAGACGAAAGCTTTGA
U. edulis       GCAAACGTAGTGGAATTCCAGACATGCAGTGTCACTTCAGACCCCAGACAAAAGCTTTGA
                * ***** ****** ******************  ******** ** ** **********

S. japonica     TCGAGAAACTCTTAGACGAGGAAATCACTCGTATAATTACTACATGTACCAATACAGTCA
S. lycidas      TCGAGAAACTCTTAGACGAGGAAATCACTCGTATAATTACTACATGTACCAATACAGTCA
S. pharaonis    TCGAGAAACTCTTAGACGAGGAAATCACACGTATAATTACTACATGTACCAATACAGTCA
S. lessoniana   TAGAGAAACTCTTAGACGAGGAAATAACTCGTATACTCACCACCTGCACCAACACTGTCA
U. edulis       TAGAGAAACTCTTAGATGAGGAAATTACTCGTATACTAACCACCTGCACCAACACCGTCA
                * ************** ******** ** ****** * ** ** ** ***** ** ****

S. japonica     ATGACATTGCAGACTTGCAGTGATACTTCA---CAGGTCAAGCAAATTCACTGGATATGC
S. lycidas      ATGACATCGCAGACTTGCAGTAATTTTTCA---CAGGTCAAGCGAATTCACTGGATATAC
S. pharaonis    ATGACATCGCAGACTTGCAGTAATTTTTCA---CAGGTCAAGCAAATTCACTGGATATAC
S. lessoniana   ATGACATCACAGACTTGCAATAA-ACTTCA---CAAGTTGAATAAATTCACCGAATGTGG
U. edulis       ATGACATTGCAGACTTGCAATAA-AAATCACAGCAGGTCAAATAAATTCGCCAAATGAGG
                *******  ********** * *    ***   ** **  *   ***** *   **

S. japonica     AACCACCAAATA--AAACTGACCAAAGCTGGAAGGCTAACATTTCACAGAAAAAGATCAA
S. lycidas      AACTACCAAAAAATAAACTGACCAAAGCTGGAATGCTAAAATTTCACAGAAAAAGACGAA
S. pharaonis    AACTACCAAA--------------------------------------------------
S. lessoniana   AACCAACAAA----AAACTGACC-AAGTTGAAATGCTAAAATTAAAAAAATAAATTTGAA
U. edulis       AACCAACAAA----AAACTGATC-AAGCTAAAATGCTAAAATTTAA--------------
                *** * ****

S. japonica     CTTTATGATTTTTAATTATTGATAAAACCAAGACAATTTCT-----------TATATATA
S. lycidas      CTTCATGATTTTGAATTATTGATAAAACCAAGACACTTTATAACATTATATATATATGTG
S. pharaonis    ------------------------------------------------------------
S. lessoniana   ACTTATGATCTTCAATTATTGATAAACCCAAAAGACTCC---------------------
U. edulis       ------------------------------------------------------------

S. japonica     TATATATATATATATATATATATATATATATATAA-----TACACACACCTGCTGAACAG
S. lycidas      TATATATATATATATATATATATATATATATATACACACTCACACTTACCTGCTGAAGAG
S. pharaonis    ------------------------------------------------------------
S. lessoniana   ----------CATATATATATATATATATATATAC-----------------TCAAACAA
U. edulis       ------------------------------------------------------------

S. japonica     AACTGTCAAAACACTGTA------ATATACAGAGCTATATATGTAGATCATAGGACTT--
S. lycidas      AACTGTCAAAACACTGTAT----TATAAACAGAGCTATATATGTAGATCATAGGATTA--
S. pharaonis    ------------------------------------------------------------
S. lessoniana   GACTGTCAGAACATTATGTATTATATATAGAGAGCTATATATGTAGATCATTGGACTATA
U. edulis       ------------------------------------------------------------

S. japonica     ----------------TATATGATATTACTTATATATTCATTATAATATCATGAAATAAA
S. lycidas      ----------------TATATGATATTACTTATATATTTATTATAATATCATGAAATAAA
S. pharaonis    ------------------------------------------------------------
S. lessoniana   TTCTAAATAGCTACTGTATATGATATTGCTTATACATTGATGCTGATATCATGAAATAAA
U. edulis       ------------------------------------------------------------

S. japonica     AAGTTAAAACTAAAAAAAAAATGGTGATTTCTGTACAAATATAAAAAAAAAAAAAAAAAA
S. lycidas      AAGTTAAAAATCTCAAAAA-----------------AAAAAAAAAAAAAAAAAAAAAAAA
S. pharaonis    --------------AAAAA-----------------AAAAAAAAAAAAAAAAAAAAAAAA
S. lessoniana   ATGTTAAAAATCCCAAAAAAA---------------AAAAAAAAAAAAAAAAAAAAAAAA
U. edulis       --------------AAAAAAA---------------AAAAAAAAAAAAAAAAAAAAAAAA
                              *****                 *** * ******************
```

Additional file 1

(A)

	Nucleotide sequence identity	Amino acid sequence identity	similarity
U. edulis	87%	92%	94%
S. japonica	82%	87%	90%
S. lycidas	81%	83%	87%
S. pharaonis	82%	82%	86%
O. vulgaris	-	69%	80%

(B)

S. lessoniana	Q	N	Y	H	F	S	N	G	W	H	P	G	G	K	R
	*	*	*	*	*	*	*	*	*	*	*	*	*	*	*
U. edulis	Q	N	Y	H	F	S	N	G	W	H	P	G	G	K	R
	*	*	*	*	*	*	*	*	*	*	*	*	*	*	*
S. japonica	Q	N	Y	H	F	S	N	G	W	H	P	G	G	K	R
	*	*	*	*	*	*	*	*	*	*	*	*	*	*	*
S. lycidas	Q	N	Y	H	F	S	N	G	W	H	P	G	G	K	R
	*	*	*	*	*	*	*	*	*	*	*	*	*	*	*
S. pharaonis	Q	N	Y	H	F	S	N	G	W	H	P	G	G	K	R
	*	*	*	*	*	*	*	*	*	*	*	*	*	*	*
O. vulgaris	Q	N	Y	H	F	S	N	G	W	H	P	G	G	K	R
	*	*	*	*	*	*	*	*	*	*	*	*	*	*	*
M. yessoensis	Q	N	F	H	Y	S	N	G	W	Q	O	G	K	R	-
	*	*		*		*	*	*	*			*			
A. californica	Q	N	Y	H	F	S	N	G	W	Y	A	G	K	K	R
	*	*	*	*	*	*	*	*	*			*		*	*

Additional file 2

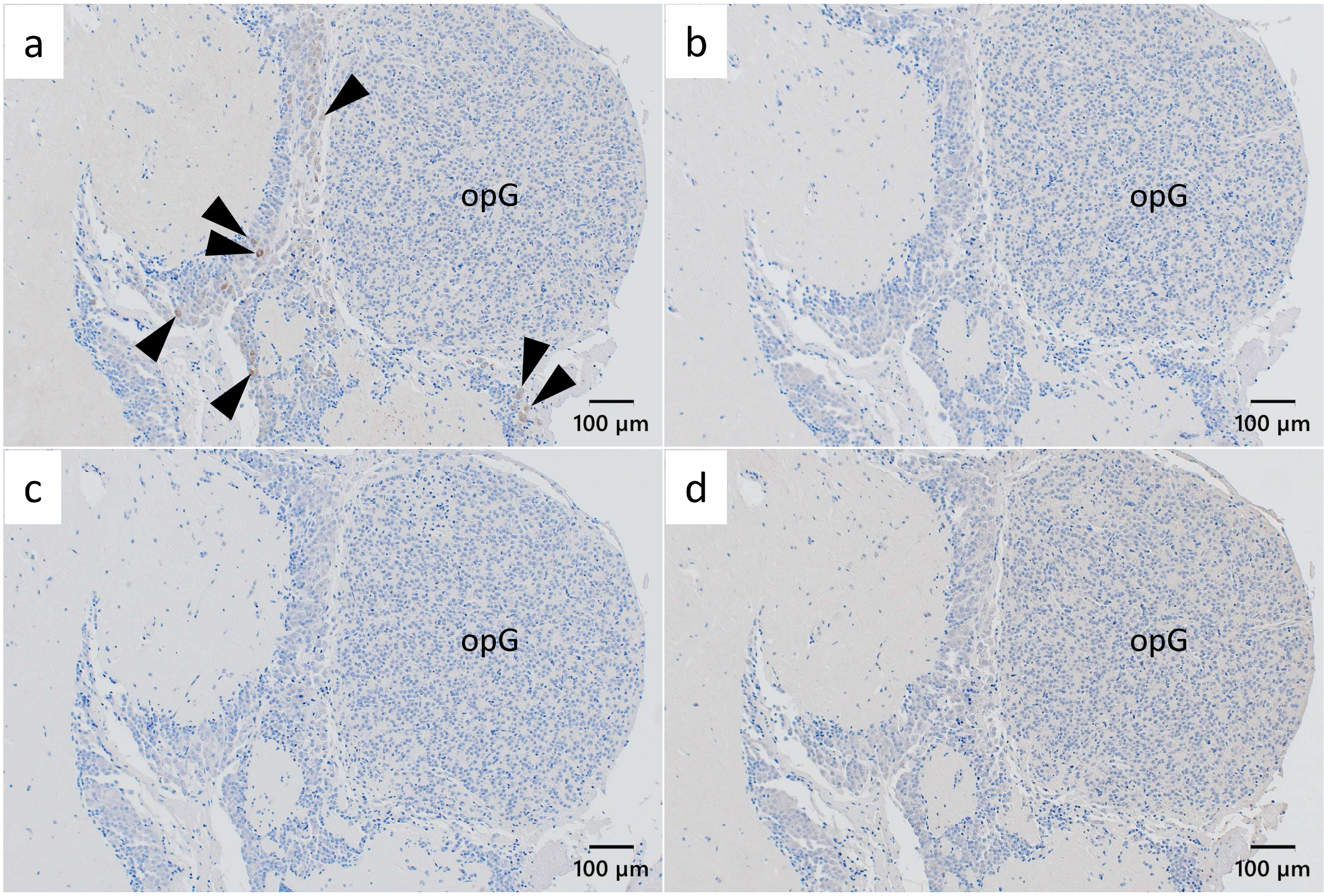

Additional file 3
